# Phylogeography of the woolly mammoth (Mammuthus primigenius) in the Minusinsk Depression of southern Siberia in the Late Pleistocene

**DOI:** 10.18699/vjgb-24-63

**Published:** 2024-09

**Authors:** S.A. Modina, M.A. Kusliy, D.G. Malikov, A.S. Molodtseva

**Affiliations:** Institute of Molecular and Cellular Biology of the Siberian Branch of the Russian Academy of Sciences, Novosibirsk, Russia Novosibirsk State University, Novosibirsk, Russia; Institute of Molecular and Cellular Biology of the Siberian Branch of the Russian Academy of Sciences, Novosibirsk, Russia; Institute of Molecular and Cellular Biology of the Siberian Branch of the Russian Academy of Sciences, Novosibirsk, Russia V.S. Sobolev Institute of Geology and Mineralogy of the Siberian Branch of the Russian Academy of Sciences, Novosibirsk, Russia; Institute of Molecular and Cellular Biology of the Siberian Branch of the Russian Academy of Sciences, Novosibirsk, Russia Institute of Archaeology and Ethnography, Siberian Branch of the Russian Academy of Sciences, Novosibirsk, Russia

**Keywords:** ancient DNA, woolly mammoth, phylogeography, mitochondrial genome, southern Siberia, древняя ДНК, шерстистый мамонт, филогеография, митохондриальный геном, Южная Сибирь

## Abstract

To date, a number of studies have been published on the phylogenetics of woolly mammoths (Mammuthus primigenius), ranging from analyses of parts of the mitochondrial genome to studies of complete nuclear genomes. However, until recently nothing was known about the genetic diversity of woolly mammoths in southern Siberia, in the Minusinsk Depression in particular. Within the framework of this effort, libraries for high-throughput sequencing of seven bone samples of woolly mammoths were obtained, two-round enrichment using biotinylated probes of modern mtDNA of Elephas maximus immobilised on magnetic microspheres and sequencing with subsequent bioinformatic analysis were carried out. Phylogenetic reconstructions showed the presence of all studied mammoths in clade I, which expanded its range. The assignment of mammoth mitotypes in the Minusinsk Depression to different clusters within clade I may indicate a sufficiently high diversity of their gene pool. Phylogeographic reconstructions revealed a genetic proximity of mitochondrial lineages of Late Pleistocene mammoths of the Minusinsk Depression and other regions of eastern Siberia and estimated their divergence time in the range of 100–150 thousand years ago, which indicates active migrations of woolly mammoths over vast territories of eastern Siberia in the late Middle Pleistoceneearly Late Pleistocene.

## Abstract (ru)

К настоящему времени опубликован ряд исследований о филогенетике шерстистых мамонтов (Mammuthus primigenius), начиная с анализа частей митохондриального генома и заканчивая изучением полных ядерных геномов. Однако до сих пор ничего не известно о генетическом разнообразии шерстистых мамонтов на Юге Сибири, в частности в Минусинской котловине. В рамках данной работы были получены библиотеки для высокопроизводительного секвенирования семи костных образцов шерстистых мамонтов, проведены двухраундное обогащение с использованием биотинилированных зондов современной мтДНК Elephas maximus, иммобилизованных на магнитные микросферы, и секвенирование с последующим биоинформационным анализом. Филогенетические реконструкции показали принадлежность всех исследованных нами вариантов мтДНК мамонтов к кладе I, что расширило ее ареал. Расположение митотипов мамонтов Минусинской котловины в разных субкладах внутри клады I может указывать на достаточно высокое разнообразие их генофонда. Филогеографические реконструкции выявили генетическую близость митохондриальных линий позднеплейстоценовых мамонтов Минусинской котловины и других регионов Восточной Сибири и их дивергенцию во временном промежутке от 100 до 150 тыс. лет назад, что свидетельствует об активных миграциях шерстистых мамонтов на обширных территориях Восточной Сибири в конце среднего–начале позднего плейстоцена.

## Introduction

The phylogeography of the woolly mammoth, one of the most important representatives of the mammoth fauna, is currently being studied on an extensive scientific basis. The Genbank database contains 32 mitogenomes of Mammuthus primigenius Blumenbach, 1799. According to palaeontological data, the common lineage of Asian elephants and woolly mammoths (Mammuthus primigenius) diverged from the lineage of African elephants (Loxodonta africana) 6 million years ago, and the divergence of the lineages of mammoths and Asian elephants (Elephas maximus) is dated by genetic data to 440 thousand–2 million years ago (Krause et al., 2006; Rogaev et al., 2006).

In 2007, the results of one of the first studies of the phylogeographic relationships of mitochondrial lineages from a large and diverse sample of woolly mammoths were published, based on mixed sequence analysis of 741 bp of the mitogenome (three genes plus part of the control region) (Barnes et al., 2007). The sample included 41 woolly mammoths from Europe, Asia (western Beringia, Kamchatka Peninsula, north-central Siberia) and North America (eastern Beringia). The samples ranged in age from 12 to 51 thousand years. The study identified two major mitogroups of woolly mammoths that existed in western and eastern Siberia, the Far East and Alaska, as well as a mitochondrial lineage of mammoths from the European region. The first mitogroup was distributed in Siberia and North America, while the second was restricted to the north of eastern Siberia, between the Lena and Kolyma river valleys. Sequence analysis of the 743 bp hypervariable region of the mitogenome of 160 mammoths from the Holarctic region of Eurasia and North America revealed 80 haplotypes forming five haplogroups (A–E), which form three major clades (A, B and C+D+E), the clustering of which is supported by high posterior probabilities. Clade A contains only Asian mitotypes, clade C contains only North American mitotypes, and the remaining haplogroups are mixed (Debruyne et al., 2008). However, the studies discussed above only focused on partial mitogenome sequences, which, unlike complete sequences, do not provide such a clear resolution of phylogeny.

Studies of 18 complete woolly mammoth mitogenomes confirmed the presence of two mitogroups in Siberia during the Late Pleistocene (Krause et al., 2006; Poinar et al., 2006; Rogaev et al., 2006; Gilbert et al., 2007). One of the clades was stably represented in the gene pool of the populations for a long time, while representatives of the second clade became extinct. The disappearance of the second clade may be related to its limited distribution (Payne, Finnegan, 2007).

There is disagreement about the timing of intraspecific divergence of mammoths. Some researchers suggest 1–2 million years ago (Gilbert et al., 2007), while others suggest around 1 million years ago based on phylogenetic reconstructions (Van der Valk et al., 2021). The representativeness of data on the diversity of mitochondrial DNA variants belonging to clades I and II is low, especially for Siberian populations; therefore, further study of local series of mtDNA samples from different regions is necessary for a complete understanding of the genetic diversity of woolly mammoths in this region.

Nuclear genome analyses have confirmed the closeness of woolly mammoths to Asian elephants (Greenwood et al., 1999; Capelli et al., 2006; Miller et al., 2008)) and estimated the time of divergence of mammoths and African elephants (Elephas maximus) at 5–6 million years ago (Poinar et al., 2006). Separate studies of nuclear genome sequences also suggest two mammoth lineages that diverged 1.5–2 million years ago (Miller et al., 2008). Whole-genome analyses, however, suggest that the split occurred between 50 and 155 thousand years ago (Palkopoulou et al., 2015). Studies of the nuclear genomes of Early and Middle Pleistocene mammoths also suggest the existence of two lineages in eastern Siberia, only one of which represents the ancestor of the woolly mammoth.

It should be emphasized that the samples studied so far from Siberia and the Far East are from the northern and eastern regions. Molecular genetic studies of samples from geographically isolated areas are revealing new genetic diversity, such as the presence of a second mitogroup of woolly mammoths in the northern part of eastern Siberia (Gilbert et al., 2007). Additional analyses of mammoth DNA samples from different regions of Siberia are allowing us to expand our understanding of the phylogenetic diversity of mammoth mtDNA and the specifics of its phylogeography. For example, a woolly mammoth genetic lineage was discovered in Taymyr that was previously thought to be characteristic only of Europe (Maschenko et al., 2017). Mammoths of southern Siberia remain understudied at the molecular genetic level, although these data are important for assessing the peculiarities of local mammoth genetic diversity and the specifics of the evolution of regional mammoth populations. To fill this gap, we studied the ancient DNA of woolly mammoths from the Minusinsk Depression.

Working with ancient DNA is challenging due to its low content, degradation and chemical changes, and possible contamination of samples by microorganisms (Pääbo et al., 2004; Brotherton et al., 2007; Carpenter et al., 2013). One of the key approaches to overcome these difficulties is the enrichment of genomic libraries with targeted DNA fragments

Hybridisation capture has a number of advantages over PCR (Meyer, Kircher, 2010; Horn, 2012). Hybridisation capture involves the preparation of a genomic library and target DNA fragments, their hybridisation and subsequent separation using magnetic particles. Hybridisation capture methods such as primer extension capture or multiplex capture of target fragments have been shown to be fast and efficient (Briggs et al., 2009; Maricic et al., 2010).

In our study, we use the enrichment method proposed by T. Maricic, M. Whitten and S. Pääbo (Maricic et al., 2010) with two rounds of hybridisation, which has been shown many times (Reich et al., 2010; Dabney et al., 2013; Thalmann et al., 2013; Vorobieva et al., 2020; Kusliy et al., 2021) to be a highly efficient approach for the analysis of the complete mitochondrial genome in ancient samples

## Materials and methods

**Fig. 1.**
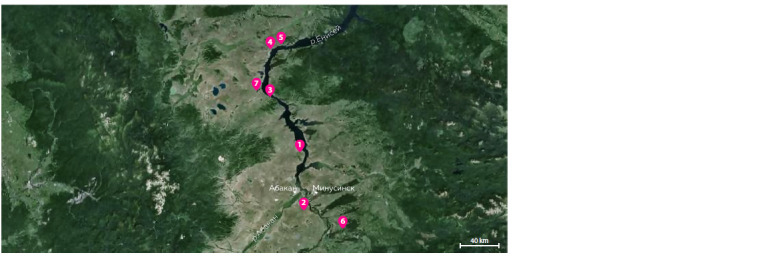
Map of locations of remains of woolly mammoths (Mammuthus primigenius) from the Minusinsk Depression. Locations (red circles) are marked with numbers that correspond to the numbers in the Table (Malikov et al., 2023).

The material for the study was collected by D.G. Malikov during expeditionary work in 2011–2021, as well as partially obtained from the collections of the Zoological Museum of the N.F. Katanov Khakass State University (ZM KSU) and the L.R. Kyzlasov Khakass National Museum of Local Lore (KNMLL). Territorially, the bone remains cover all parts of the Minusinsk Depression (Fig. 1) and come from six localities of different geological age (see the Table).

**Table 1.**
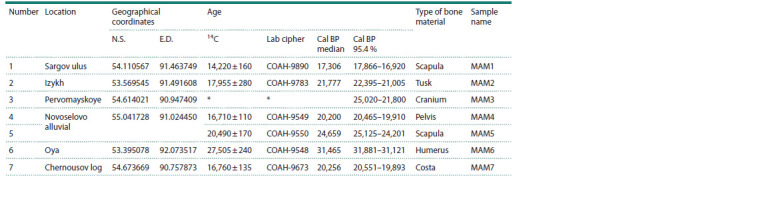
Information on bone material * Data obtained from other samples from the collection.

14C dates were obtained for all samples (except MAM3) and were previously published in a summary (Malikov et al., 2023). Dating was performed at the Laboratory of Cenozoic Geology, Palaeoclimatology and Mineralogical Indicators of Climate, V.S. Sobolev Institute of Geology and Mineralogy (IGM) SB RAS by the benzene scintillation method. For specimen MAM3 from the Pervomayskoye locality, the age was determined on the basis of 14C dates obtained from other M. primigenius remains from this locality with similar preservation of bone material. For radiocarbon dating and DNA extraction, different parts of the same bone remains were used, which were not pre-treated with chemical reagents.

The isolation of ancient DNA from bone powder was performed according to the protocol described in the article by H. Yang et al. (Yang et al., 1998). Оbservance of all criteria of purity and authenticity of the DNA samples obtained (Gilbert et al., 2007). As part of this work, we obtained mitogenomic libraries for high-throughput sequencing from seven woolly mammoths from the Minusinsk Depression (southern Siberia), 17–30 thousand years old, using the TruSeq Nano Library Prep Kit (Illumina) according to the manufacturer’s protocol. For these libraries, we performed a two-round enrichment by hybridisation with biotinylated fragments of modern mitochondrial DNA from Elephas maximus L., 1758, immobilised on Dynabeads® Streptavidin magnetic particles (Life Technologies, USA), which allowed us to significantly increase the proportion of endogenous ancient mitochondrial DNA.

## Results

The characteristics of seven sequences of mitogenomes of Late Pleistocene woolly mammoths from the Minusinsk Depression studied by us are presented in the summary table (https://docs. google.com/spreadsheets/d/1XaSB-cb14rxNy0aas5xDLUiI_ YiKeBSy-_Kwe1Rt2KQ/edit?usp=sharing). The average depth of coverage of the characterised mitogenomes varies from 0.5 to 15.5x, and the width of coverage ranges from 38 to 99.5 % of the reference mitogenome length. The average percentage of uniquely mapped pooled reads to total pooled reads is 7.9 %. Based on the values of base deamination frequency and average size of DNA fragments obtained, we can conclude that the mammoth bone samples from the Minusinsk Depression have a high degree of DNA preservation, most likely due to relatively good environmental conditions for DNA preservation.

**Fig. 2.**
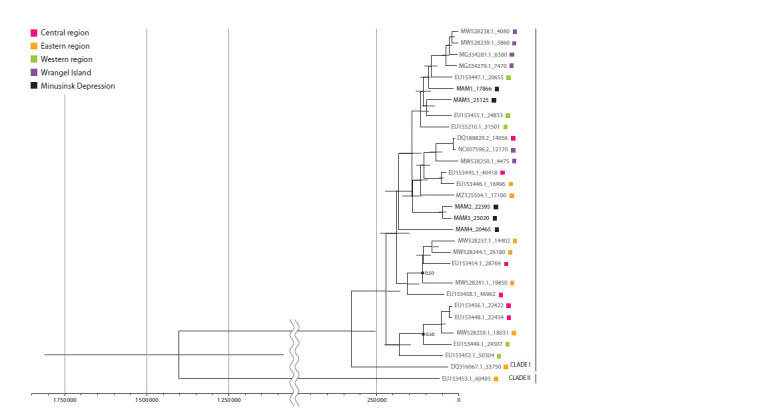
BEAST phylogenetic reconstructions based on mtDNA sequences of five woolly mammoths from the Minusinsk Depression and 25 previously published mtDNA sequences of woolly mammoths from the Genbank database The phylogenetic tree was constructed using the BEAST software platform with internal calibration of branch divergence time based on radiocarbon dating of samples. The colours of the squares reflect the geographical origin of the samples: Central region – part of Siberia washed by the Kolyma and Lena rivers; Eastern region – part of Siberia east of the Kolyma River; Western region – part of Siberia east of 70° E and west of the Lena River; Wrangel Island – a group of islands in northeastern Siberia. The Bayesian posterior probability of the tree topology is greater than 0.75 in all cases except where this is indicated as numbers next to the tree nodes. The light grey lines through the tree nodes denote the standard deviation of the median estimates of divergence times. The radiocarbon dating of each specimen is given next to the name of each specimen after the “_” sign.

Only specimens with sufficient breadth (more than 70 %), depth of mitogenome coverage (more than 2) and radiocarbon dates were used to construct a phylogenetic tree with a certain time of divergence of genetic lineages (Fig. 2). These criteria were met by five of the seven specimens examined. The same selection criteria were used to include sequences from previously published woolly mammoth mitogenomes in the analysis. The analysis was performed using the BEAST software platform, based on the topology of a constructed tree with an uncertain time of divergence of genetic lineages

The divergence of the genetic lineages of woolly mammoths from the Minusinsk Depression from the most genetically similar mammoths from other regions of eastern Siberia occurred in the time interval between 150 and 100 thousand years ago. Woolly mammoths from the Minusinsk Depression form sister clades with woolly mammoths from other represented regions of Siberia (Wrangel Island, central, western and eastern regions), which distinguishes them from some other studied local groups of mammoths, such as the mammoths from Wrangel Island, which were in a stage of reduced genetic diversity.

## Discussion

This study allows us to estimate the mitochondrial genetic diversity of woolly mammoths in the Minusinsk Depression. The phylogenetic reconstruction obtained shows that the divergence of two clades of woolly mammoths occurred 1–2 million years ago, which correlates with the results of studies of complete mammoth mitochondrial and nuclear genomes described in the introduction (Gilbert et al., 2008; Miller et al., 2008). The divergence of the genetic lineages of mammoths from the Minusinsk Depression and the most genetically similar genetic lineages of mammoths from other regions of eastern Siberia occurred in the time interval from 150 to 100 thousand years ago. The structure of the phylogenetic tree we constructed indicates that the mtDNA sequences of woolly mammoths from the Minusinsk Depression do not form a separate clade on the tree, but are dispersed in different clusters of clade I. At the same time, mtDNA sequences of mammoths from the Minusinsk Depression form sister clades with mtDNA sequences of woolly mammoths from other represented regions of Siberia (Wrangel Island, central, western and eastern regions), which may indicate intensive mammoth migrations across large areas of eastern Siberia in the late Middle to early Late Pleistocene.

The placement of mitotypes of Late Pleistocene mammoths from the Minusinsk Depression in different clades within clade I, in contrast to Holocene mammoths from Wrangel Island, indicates a low probability that they were on the verge of extinction during this period. At this stage, we propose two possible explanations for their position on the phylogenetic tree: 1) the samples studied belong to a single (permanent in the region) population of mammoths characterised by high phylogenetic diversity of mtDNA; 2) the samples studied were obtained from representatives of different mammoth populations that migrated independently through the Minusinsk Depression during the Late Pleistocene. At this stage we have arguments “for” and “against” each of the versions. For example, the fact that some of the specimens from localities of different geological age form either single or closely related clades (Fig. 2), regardless of their geological age and location, may support the idea that the mammoths of the Minusinsk Depression studied by us belong to a single population. In addition, the revealed isotopic signal of carbon and nitrogen stable isotopes (δ13C and δ15N) in mammoths from the Minusinsk Depression differs significantly from those in northern populations of the species (Malikov et al., 2023). This suggests that the animals lived in this region for a relatively long time, which is reflected in their isotopic indices.

At the same time, the wide dispersal of the mammoths studied on the general phylogenetic tree may indicate that they belonged to different populations. In support of this version, it should be noted that there are currently no mammoth finds in the region under consideration that can be confidently attributed to warm Late Pleistocene. It is possible that during the warm intervals of the Late Pleistocene, conditions in southern Siberia were unfavourable for the permanent habitat of M. primigenius. In this case, representatives of the species could only repeatedly migrate into the depression during cold periods. Furthermore, modern African elephants are known to live in small groups of 6–8 individuals with seasonal home ranges of 130–1,600 km2 (Nasimovich, 1975). However, under unfavourable conditions, individual elephant movements can reach 32,000 km2 per year (Wall et al., 2013). The total area of the Minusinsk intermountain trough (including the Nazarovskaya Depression) is approximately 100,000 km2 (Vorontsov, 2012). The maximum length of the Minusinsk Depression in the northwestern direction is about 450 km, with a maximum width (along the southern Minusinsk trough) of ~400 km. Consequently, the total area of the region is only sufficient to support a small population of large animals such as mammoths. This suggests that the area of the Minusinsk Depression is insufficient to support permanent populations of M. primigenius. This is because the resource base of the depression is limited and the annual seasonal migrations of the species are comparable to or exceed the size of the depression itself.

Another argument for the migratory nature of the Minusinsk Depression mammoth population is the fact that two samples from the Novoselovo alluvial site (MAM4 and MAM5) showed maximum genetic distance (Fig. 2). On the contrary, samples from the Pervomayskoye (MAM3) and Izykh (MAM2) localities formed a single group. Although the sites are more than 100 km apart, they date to approximately the same time interval (about 21.8 thousand years ago). It is possible that these individuals belong to a single population that migrated into the region from time to time, possibly over a long period of time.

If the second concept is true, the data obtained can be regarded as confirmation of the local extinction of mammoths in the Minusinsk Depression at the Pleistocene-Holocene boundary, which was probably caused by the development of taiga and forest-steppe landscapes in western and eastern Siberia. As a result, the replenishment of populations of herbivorous mammals of the Minusinsk Depression and their seasonal migrations stopped (Malikov, 2015).

## Conclusion

In summary, climate change from the Late Glacial to the Holocene resulted in a reduction of open areas in Eurasia, which in turn reduced the habitat area of mammoths and other steppe animals. This process involved complex changes in climate and vegetation in space and time, the survival of species in refugia, local extinctions and temporary expansion of habitats

One of the most effective approaches to a detailed reconstruction of these processes is the study of local series of mammoth mitochondrial DNA samples belonging to different chronological periods. Our study is a step in this direction

It is necessary to continue palaeontological and molecular genetic studies of woolly mammoths in isolated regions of Siberia in order to fully determine their genetic diversity and the causes of their extinction in this locality. It is preferable to study complete genomes, which will make phylogeographic analyses more accurate and reliable.

## Conflict of interest

The authors declare no conflict of interest.
